# Directed dihydroxylation of a poly(cyclooctadienol) toward densely-hydroxylated polyol adhesives†

**DOI:** 10.1039/d5py00044k

**Published:** 2025-04-07

**Authors:** Lauren S. Cooke, Aleksandr V. Zhukhovitskiy

**Affiliations:** a The University of North Carolina at Chapel Hill, Department of Chemistry Chapel Hill NC 27514 USA alexzhuk@email.unc.edu

## Abstract

Adhesives play an important role in an array of industries, including construction, medicine, paper products, and more. Specialty applications are expanding and evolving to require more niche adhesives to suit unique needs. In one of its many applications, poly(vinyl alcohol) (PVA) serves as a water-soluble adhesive; yet, PVA's adhesive ability is ultimately constrained by its fixed degree of hydroxylation and head-to-tail construction. In this work, we synthesize a water-soluble polyol that features a higher density of hydroxylation than PVA and exhibits thermal and adhesive properties comparable to PVA and Elmer's® clear glue. We do so *via* catalytic dihydroxylation of a polybutadiene derivative formed *via* ring-opening metathesis polymerization of a cyclooctadiene with a single allylic alcohol. Notably, such global catalytic dihydroxylation relies on allylic and homoallylic hydroxyl moieties as directing groups, and as such, could not previously be implemented in the context of unfunctionalized polybutadiene. Hence, this work provides an alternative entry to polyols with high alcohol group content toward water-soluble specialty adhesives.

## Abstract 2

Directed dihydroxylation of an unsaturated polymer yields polyols with a dense array of alcohol groups toward applications in adhesives.

## Graphical abstract

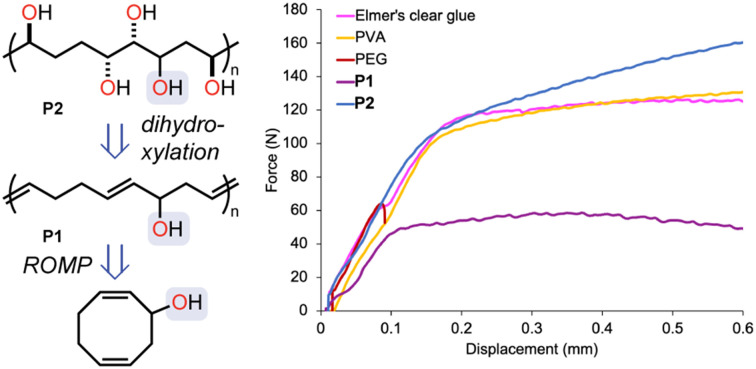

From aircraft to masking tape, polymer-based adhesives are indispensable in a variety of modern technologies.^1^ The demand for new specialty adhesives is ever-increasing, with a need for desirable properties that align with future applications, such as sustainable glues or adherents in electric vehicles.^2,3^ Polyvinyl alcohol (PVA) is one such industrial adhesive utilized in manufacturing, packaging, medicine, and numerous other applications.^4^ PVA is synthesized from the hydrolysis of polyvinyl acetate (PVAc), derived from the atactic head-to-tail addition of vinyl acetate monomers^5^*via* free radical polymerization. The 1,3-alcohol structure is maintained consistently throughout the hydrolyzed polymer.^6^ To understand the effect of polyol structure on adhesion, we developed a densely-functionalized polyol P2, and compared its thermal and adhesive properties to PVA and Elmer's® clear glue. Our goal of synthesizing P2 motivated the development of a new method for generating polymeric 1,2-diols *via* directed dihydroxylation of an unsaturated polymer.

To access such densely hydroxylated polyols, a different synthetic strategy would be required compared to the one utilized to make PVA. In particular, direct dihydroxylation of a formal polybutadiene derivative prepared *via* ring-opening metathesis polymerization (ROMP) of cyclooctadienol M1 (Fig. 1) appeared to be a promising route. Indeed, Upjohn dihydroxylation using catalytic osmium tetroxide^7^ had been demonstrated in the context of poly(oxa-norbornene)s and poly(norbornene)s prepared *via* ROMP;^8–12^ however, this approach has previously failed in the context of polybutadiene^13,14^ and is almost non-existent in poly(cyclooctadiene) derivatives, save for a reference in one thesis.^15^ The key challenges encountered in the dihydroxylation of polybutadiene are phase separation and poor polymer solubility. We hypothesized that pre-installed (homo-)allylic hydroxyl groups would both improve the polymer solubility in the polar reaction media common to Upjohn dihydroxylation and serve as directing groups that could accelerate reactivity. Reports of allylic alcohols undergoing osmium-catalyzed dihydroxylation support the directing ability of hydroxyl groups, resulting in highly-hydroxylated compounds.^16^ Thus, we imagined an analogous reactivity profile to previous reports,^17^ where directed dihydroxylation could be applied to unsaturated polymers by incorporating (homo-)allylic alcohols.

**Fig. 1:**
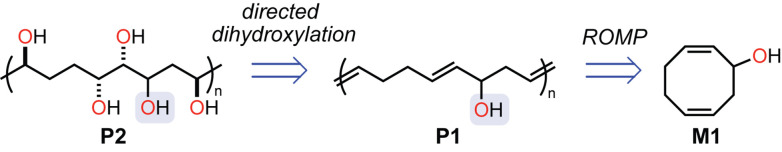
Retrosynthetic analysis applied to polyol P2.

Synthesis of the densely-hydroxylated polyol P2 was successfully carried out in three steps (Fig. 2A). First, M1 was produced *via* Riley oxidation^18,19^ of 1,5-cyclooctadiene (COD). Next, a version of the 3^rd^-generation Grubbs catalyst^20–22^ was employed for the ROMP of M1 to form the partially-hydroxylated unsaturated polymer P1 as a brown rubbery solid (Fig. 2A). Notably, in the ^1^H nuclear magnetic resonance (NMR) spectrum of P1, we observe two distinct methine resonances at 4.07 ppm and 4.41 ppm with nearly equal integration (Fig. 2B), which suggests random head-to-head and head-to-tail linking of successive monomers and/or ring-opening at both alkenes of M1. Complete dihydroxylation (>95%) of P1 was confirmed using ^1^H NMR analysis which revealed the total disappearance of alkene resonances (Fig. 2B). Gel permeation chromatography with multi-angle light scattering (GPC-MALS) with THF as the eluent was utilized to determine the number-average molecular weight (*M*_n_) and dispersity (*Đ*) of different batches of P1 produced by varying the monomer-to-initiator ratio (Fig. S1 and S2†): *M*_n_ = 86.7 kg mol^−1^ and *Đ* = 2.37 for one batch, and *M*_n_ = 163 kg mol^−1^ and *Đ* = 2.41 for another. Additionally, molecular weight control was achievable by incorporating simple *cis*-alkenes as chain transfer agents,^23,24^ reducing metathesis catalyst loading (Table S1†). Lastly, OsO_4_-catalyzed dihydroxylation of both batches of P1 was performed to afford polyol P2 in good yield (82%) as a white material with a cotton-like appearance (Fig. 2A). Residual osmium content is estimated to be 8.6 μg mg^−1^ for one batch of P2, and 1.3 μg mg^−1^ for another using inductively coupled plasma mass spectrometry (ICP-MS), corresponding to an 82% and 97% removal of osmium from the material post-workup. For the remainder of the manuscript we focus on P2 derived from the first batch of P1 (*M*_n_ = 86.7 kg mol^−1^), as this molecular weight value more closely reflects the PVA control (89–98 kg mol^−1^).

**Fig. 2:**
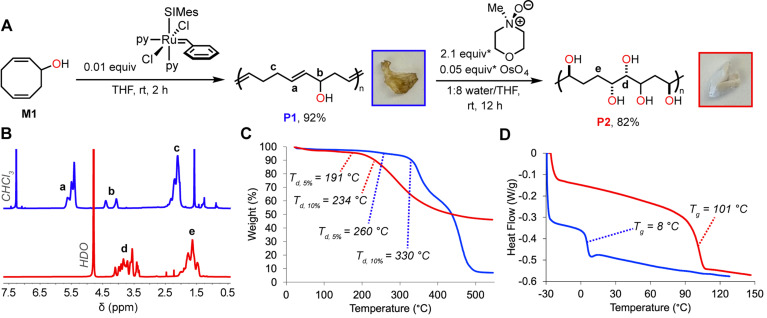
(A). Reaction scheme and photographs for synthesis of P2. Representative ^1^H NMR spectra (B), TGA (C), and DSC plots (D) for P1 (10 °C min^−1^; *M*_n_ = 86.7 kg mol^−1^) and P2 (20 °C min^−1^). *Equivalents calculated and reported relative to P1 repeat unit.

With P1 and P2 in hand, we performed thermogravimetric analysis (TGA) and differential scanning calorimetry (DSC) of these materials to evaluate their thermal properties. P1 (86.7 kg mol^−1^) had a temperature of decomposition at 5% weight loss (*T*_d, 5%_) of 260 °C and *T*_d, 10%_ of 330 °C at 10% weight loss, while the corresponding P2 had a *T*_d, 5%_ of 191 °C and *T*_d, 10%_ of 234 °C (Fig. 2C). Notably, P2 retained a significant amount of charred material and exhibited a total weight loss of about 54%, even when heated to 550 °C. The glass transition temperature (*T*_g_) of P1 is 8 °C, while the *T*_g_ of P2 is considerably higher at 101 °C (Fig. 2D). This dramatic difference in *T*_g_ is expected: more thermal energy is required to impart chain mobility with increased inter- and intra-chain hydrogen bonding.^25^ Notably, the *T*_g_ of P2 is even higher than that of PVA (reported in the 75–85 °C range);^26^ yet, in further contrast with PVA,^27^P2 exhibits no melting behavior *via* DSC, suggesting amorphous morphology, although this feature may be absent because of lower-temperature degradation: P2 undergoes degradation (*T*_d, 5%_ = 191 °C) before PVA's reported melting temperature (>220 °C).^28^

Having established the effects of dense backbone hydroxylation on thermal properties, we sought to explore its effects on adhesion using the lap shear test. As our controls, we tested a similar batch of P1 (92.2 kg mol^−1^, Fig. S3†), poly(ethylene glycol) (PEG) (20 kg mol^−1^), PVA (89–98 kg mol^−1^), and commercial Elmer's® clear glue (an aqueous emulsion of PVAc, PVA, and propylene glycol).^29^ As our substrate, we selected glass slides, because their hydrophilic surfaces are wetted well by the aqueous solutions of the polar polymers. The adhered samples were prepared by applying approximately the same amount of adhesive by weight in solution to a surface area of 635 mm^2^ (1 in by 1 in) and clamping the two surfaces together using binder clips (Fig. 3A). After allowing the samples to dry for several days, they were further annealed in a vacuum oven around each adhesive's *T*_g_ for 3 hours to minimize macroscopic inhomogeneity in the adhesive film between the glass substrates.

**Fig. 3:**
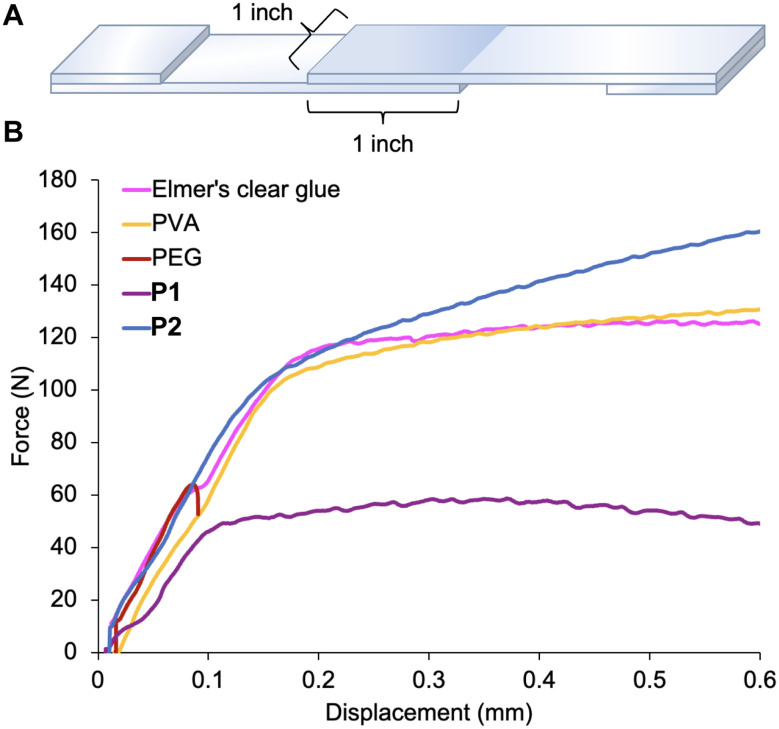
(A). Illustration of a typical sample for analysis. (B). Plot of force (N) *versus* displacement (mm) for a representative sample closest to the average for the adhesive.

Lap shear tests were performed by clamping both ends of the adhered substrates (Fig. 3A) and pulling at a constant strain rate (0.05 mm s^−1^) in opposing directions. Approximately 10 replicates were tested for each sample, and the representative results are plotted in Fig. 3B and summarized in Table 1. Good agreement was observed among the P2, P1, PVA, and Elmer's® clear glue replicates before the yield point (Fig. S4–S7†), while PEG exhibited a broader distribution of yield strength values (Fig. S8†).

**Table 1:** Average adhesive data from lap shear tests. Yield strength values were calculated by dividing the yield force (N) by the adhered area of the samples (635 mm^2^)

Adhesive	Yield strength (MPa)
PEG	0.10 ± 0.04
PVA	0.16 ± 0.01
Elmer's	0.18 ± 0.01
P1	0.0750 ± 0.002
P2	0.17 ± 0.02

The yield strength measured for P2 (0.17 ± 0.02 MPa) proved to be comparable to PVA (0.16 ± 0.01 MPa) and Elmer's® clear glue (0.18 ± 0.01 MPa). Additionally, P2 outperforms PEG (0.10 ± 0.04 MPa) in yield consistency and failure mode—PEG is brittle, while P2 exhibits more plastic behavior. P1 demonstrated poor adhesive performance (0.0750 ± 0.002 MPa), displaying creep-like behavior when pulled, evident both visually and in a steady decline in measured force over greater displacements (Fig. S4†). These results indicate that P2 performs similarly to PVA and Elmer's® clear glue; additionally, increasing degree of hydroxylation from P1 to P2 corresponds to an over two-fold increase in adhesive strength.

Spanning a variety of industries, adhesives play a critical role in our modern world. PVA is one such polyol adhesive with commercial significance, and we hypothesized that tuning polymer structure and hydroxyl group density would directly impact adhesive and thermal properties. In conclusion, we developed a highly-hydroxylated polyol P2 that could serve as an adhesive for specialty applications where moderate strength, water-solubility, and thermal stability are desirable. P2 performed well when compared to its adhesive counterparts and significantly outperformed its less decorated precursor P1. Based on literature precedents, we installed (homo-)allylic hydroxyl directing groups to impart both improved solubility to the polymer and to accelerate dihydroxylation which had previously not been observed with hydroxyl-free polybutadiene.

## Author contributions

The idea for this work was conceived by A.V.Z. All experiments and analysis were performed by L.S.C., and A.V.Z. supervised this work. This manuscript was written and revised through contributions of both authors, with approval given to the final version of this document.

## Conflicts of interest

There are no conflicts to declare.

## Supplementary Material

PY-016-D5PY00044K-s001

## Data Availability

The data supporting this article have been included as part of the ESI.†
