# Supplementary material for: Directed dihydroxylation of a poly(cyclooctadienol) toward densely-hydroxylated polyol adhesives
Source: Polym Chem. 2025 Apr 7;16(18):2128–32. doi: 10.1039/d5py00044k (PMC12679346; doi:10.1039/d5py00044k)
Supplement: PY-016-D5PY00044K-s001 [file PY-016-D5PY00044K-s001.pdf]

This journal is © The Royal Society of Chemistry 2025

# Supplementary Information for

**Directed dihydroxylation of a poly(cyclooctadienol) toward densely-hydroxylated polyol adhesives**

Lauren S. Cooke[1], Aleksandr V. Zhukhovitskiy*,[1]

[1]Department of Chemistry, University of North Carolina – Chapel Hill, Chapel Hill, North Carolina 27599, United States

Correspondence to: alexzhuk@email.unc.edu

## Table of contents


MATERIALS AND METHODS .......... 3

PURCHASED MATERIALS .......... 3
NUCLEAR MAGNETIC RESONANCE (NMR) SPECTROSCOPY .......... 3
GEL PERMEATION CHROMATOGRAPHY (GPC) .......... 3
THERMOGRAVIMETRIC ANALYSIS (TGA) .......... 4
DIFFERENTIAL SCANNING CALORIMETRY (DSC) .......... 4
INDUCTIVELY COUPLED PLASMA MASS SPECTROMETRY (ICP-MS) .......... 4
ADHESIVE SAMPLE PREPARATION/TENSILE TESTING .......... 4
GENERAL SYNTHETIC PROTOCOLS .......... 5
MATERIALS PREPARED FOLLOWING REPORTED OR MODIFIED PROCEDURES .......... 5
MATERIALS PREPARED USING NEW PROCEDURES .......... 5

SUPPLEMENTARY FIGURES .......... 7

FIGURE S1. .......... 7

FIGURE S2. .......... 8

FIGURE S3. .......... 9

FIGURE S4. .......... 10

FIGURE S5. .......... 11

FIGURE S6. .......... 12

FIGURE S7. .......... 13

FIGURE S8. .......... 14

SUPPLEMENTARY TABLES .......... 15

TABLE S1. .......... 15

TABLE S2. .......... 16

TABLE S3. .......... 17

TABLE S4. .......... 18

TABLE S5. .......... 19

TABLE S6. .......... 20

SPECTRAL DATA .......... 21

FIGURE S9. .......... 21

FIGURE S10. .......... 22

FIGURE S11. .......... 23

FIGURE S12. .......... 24

FIGURE S13. .......... 25

FIGURE S14. .......... 26

## Materials and methods

### Purchased materials

Cyclooctadiene (COD) was purchased from Sigma-Aldrich in a Sure-Seal™ bottle and used without further purification. Selenium dioxide ($SeO_2$) was purchased from Thermo Scientific, pyridine was purchased from Sigma-Aldrich, *N*-methylmorpholine *N*-oxide (NMO) was purchased from Beantown Chemical, and 2nd generation Grubbs catalyst (G2) was purchased from Sigma-Aldrich; all were used without further purification. Osmium tetroxide ($OsO_4$) was purchased as a 4 wt% solution in water from Sigma-Aldrich, and was used without further purification. Poly(ethylene glycol) (PEG, 20 kg/mol) was purchased from EMD Millipore Corporation, poly(vinyl alcohol) (PVA, 89–98 kg/mol) was purchased from Aldrich, and Elmer's® clear glue was purchased from a local convenience store; all materials were used as received.

Solvents were purchased from VWR, Sigma-Aldrich, Fisher, or Koptec, and were used as they were received. If utilized in air-sensitive experiments, they were sparged with argon and passed through purification columns prior to use.[1] Solvents used in moisture-sensitive experiments were degassed using the freeze-pump-thaw method and subsequently dried over activated 3Å molecular sieves before being stored in a nitrogen-filled glove box. Deuterated solvents were purchased from Cambridge Isotope Laboratories, Inc. and were used as they were received.

Silica gel (SiliaFlash® F60) (230–400 mesh, 40–63 micron particle size, 60 Å pore size) used for purification and glass-backed silica gel plates 20 x 20 $cm^2$ (SiliaPlate™ TLC Plates Optimized for $KMnO_4$, 250 µm, F254) for thin-layer chromatography were both purchased from Silicycle.

### Nuclear magnetic resonance (NMR) spectroscopy

$^1$H and $^{13}$C NMR spectra were collected using Bruker NMR spectrometers operating at 400, 500, 600, and 850 MHz for $^1$H (100, 125, 150, and 213 MHz for $^{13}$C, respectively). These instrument models with the corresponding supporting federal grants are as follows: Bruker AVANCE II3I Nanobay 400 MHz (NSF Grant No. CHE-0922858), Bruker AVANCE III 500 MHz (NSF Grant No. CHE-0922858), Bruker AVANCE III 600 MHz (NSF Grant No. CHE-0922858), Bruker AVANCE NEO 600 MHz (NSF Grant No. CHE1828183), and Bruker Avance III HD 850 MHz (NCI of NIH Grant No. P30CA016086). Chemical shifts are expressed in parts per million (ppm), with splitting patterns designated as s (singlet), d (doublet), t (triplet), q (quartet), sept (septet), oct (octet), m (multiplet), b (broad), and combinations thereof. Scalar coupling constants $J$ are reported in Hertz (Hz). MestReNova v14.1.1-24571 software (Mestrelab Research S. L.) was used for spectral analysis. $^1$H and $^{13}$C NMR spectra were referenced to residual monoproteo-solvent peaks as reported in literature.[2]

### Gel permeation chromatography (GPC)

Analytical GPC (for organic-soluble polymers) was performed on an Agilent Technologies 1260 Infinity II instrument equipped with two PL gel 10 µm mixed-B LS columns connected in series, a Wyatt Technologies DAWN 8-angle light scattering (MALS) detector (l = 658 nm), a Wyatt Technologies ViscoStar differential viscometer, and a Wyatt Technologies Optilab T-rEX differential refractometer. Tetrahydrofuran (THF) (35 °C) served as the eluent. The samples were prepared by dissolution of the polymer samples at a concentration of ~1 mg/mL in HPLC-grade

THF stabilized with butylated hydroxytoluene (BHT), followed by filtration through a 0.22-µm syringe filter. The dn/dc values were calculated for purified polymers in Astra software using the 100% mass recovery method, and an average value of 0.12 was used for calculating **P1** data.

**Thermogravimetric analysis (TGA)**
This analysis was carried out using a TA Instruments TGA5500 Thermogravimetric Analyzer. Characterization was performed under nitrogen using aluminum pans with a heating rate of 20 °C/min from room temperature to 550 °C. Values for the decomposition onset temperature were obtained from weight percent vs temperature (°C) plots.

**Differential scanning calorimetry (DSC)**
This analysis was carried out using a TA Instruments DSC250 Differential Scanning Calorimeter using aluminum pans, under the flow of nitrogen, with a heating and cooling rate of 10 °C/min for polyene, **P1**, and 20 °C/min for polyol, **P2**. The glass-transition temperatures ($T_g$) were obtained from the second heating scan.

**Inductively coupled plasma mass spectrometry (ICP-MS)**
This analysis was operated in standard mode, with all operating parameters optimized to meet requirements as defined by the manufacturer prior to method calibration and analysis. Calibration curves were constructed using a zero-point standard and a six-point calibration curve in a range 2 to 100 ppb. Five replicates were analyzed per sample. Quantification was performed with osmium (Os) as an internal standard. The samples were prepared in 2% hydrochloric acid solution and diluted with 2% nitric acid for analysis. Dilution was adjusted so the samples' measured concentration was within the calibration range. Percent osmium removal was calculated by creating a ratio of initial osmium content in the material and dividing that value by measured osmium content in the material post-workup. Initial moles of osmium were converted to micrograms, and this value was divided by the theoretical yield of **P2** and the total amount of $OsO_4$ added; this value is the starting µg Os/mg material. See the equation below for clarity.

$$Os_{initial} = \frac{X_{\mu g,Os}}{Y_{mg,P2\ theor.} + Z_{mg,OsO_4}}$$

$Os_{final}$ from ICP-MS analysis

$$\%_{Os,removed} = 100 - \left(\frac{Os_{final}}{Os_{initial}} * 100\%\right)$$

**Adhesive sample preparation/tensile testing**
All samples for tensile testing were prepared by deposition of ~10 mg of sample in 0.1 mL solution (water as solvent for PVA, PEG, **P2**, Elmer's® clear glue and THF for **P1**) onto a Fisherbrand™ economy plain glass microscope slide. Another glass slide was then placed onto the adhesive, and the two slides were clamped together using binder clips for 48 hours. Further processing was performed in a vacuum oven for 2 hours, with 90 °C for the **P2** & PVA samples and 30 °C used for the Elmer's® clear glue and **P1** samples. The glass slides were weighed before and after this process, giving the final weight of adhesive applied as used in yield strength per weight calculations. Uniaxial tensile testing was performed on specimens at a strain rate of 0.05 mm/s on an Instron 34SC-1 Single Column Universal Tester. Pneumatic grips were used

with a grip pressure of 4.50 bar. Yield strength values were determined by plotting lines of best fit onto linear regions before and after the transition, and then evaluating their intersection point.

**General synthetic protocols**

All air-sensitive reactions were carried out in flame- or oven-dried glassware in a nitrogen filled glove box or using standard Schlenk techniques. Inhomogeneous reaction mixtures were stirred with Teflon-coated magnetic stir bars. Reactions were monitored via NMR and TLC, and the TLC plates were visualized under UV irradiation or via standard staining procedures. Removal of solvents in vacuo was achieved using an IKA rotary evaporator and a Schlenk line (~20 mTorr, dynamic vacuum). Purification via flash chromatography was carried out following standard procedures.

**Materials prepared following reported or modified procedures**

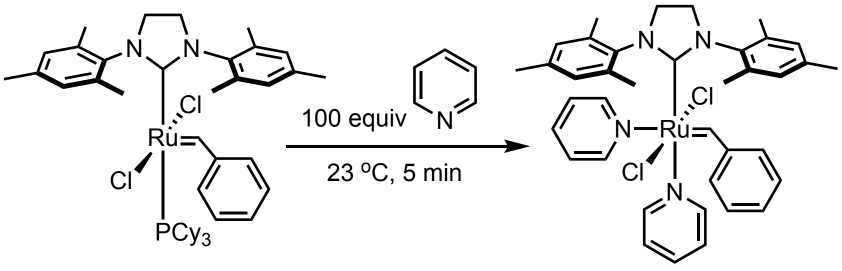


**G2** **G3**

**G3** (350 mg, 81.8%) was synthesized as reported, except that the reaction was performed in pyridine alone in the absence of solvent. The product was collected as a green precipitate from pentane. $^{1}$H NMR spectrum matched the reported one.[3]

**Materials prepared using new procedures**

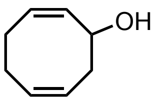


**(2*Z*,6*Z*)-cycloocta-2,6-dien-1-ol**

Under ambient conditions, $SeO_2$ (1.03 g, 9.24 mmol, 2.00 equiv) solid was added to a 250 mL three-necked flask fitted with a reflux condenser, followed by ethanol (46 mL). The heterogenous solution was stirred vigorously at room temperature, and COD (0.57 mL, 4.62 mmol, 1.00 equiv) was added to the flask via syringe. The solution was heated to 100 °C and allowed to reflux for ~48 hours. Upon completion, the reaction mixture was cooled to room temperature and extracted with chloroform ($CHCl_3$) (50 mL), washed with saturated sodium bicarbonate ($NaHCO_{3(aq)}$) (50 mL) solution, followed by brine (50 mL) and de-ionized (DI) water (50 mL). The organic layer was then collected, dried over sodium sulfate ($Na_2SO_4$) granules, and filtered. The filtrate was concentrated in vacuo and sparged with nitrogen, yielding crude product as an orange oil. The pure product was isolated using flash chromatography on silica gel using 8:2 hexanes: ethyl acetate ($R_f$ = 0.30), isolated as a pale-yellow oil (84.8 mg, 14.8%).

$^{1}H$ NMR (400 MHz, $CDCl_3$): δ 5.66–5.49 (m, 4H), 4.89 (ddd, 1H, J = 4.6 Hz), 2.78–2.69 (m, 1H), 2.53–2.33 (m, 3H), 2.31–2.09 (m, 2H). $^{13}C$ NMR (150 MHz, $CDCl_3$): δ 133.5, 130.0, 127.9, 125.6, 70.2, 36.9, 28.7, 27.6 ppm.

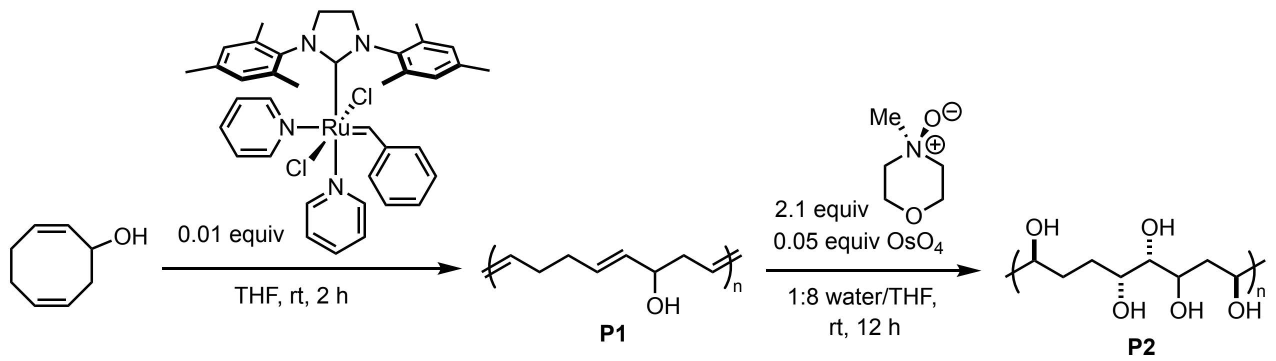


**Poly(cycloocta-2,6-dien-1-ol), P1**

In a nitrogen-filled glovebox, (2*Z*,6*Z*)-cycloocta-2,6-dien-1-ol **M1** (20.0 mg, 0.16 mmol, 1.00 equiv) was dissolved in THF (55 μL) in a small vial with a stir bar. **G3** (1.2 mg, 1.6 μmol, 0.01 equiv) was dissolved in THF (55 μL) in a separate vial. In one quick addition, the solution of G3 was added to the stirring reaction solution, and the mixture was allowed to stir for 2 hours at 23 °C. The reaction vial was removed from the glovebox. Ethyl vinyl ether (EVE) (100 μL) and THF (0.5 mL) were added, and the solution was stirred for 10 minutes. The reaction mixture was added dropwise to a vigorously stirring, cold solution of pentanes (15 mL). After decanting pentanes, the pure polymer was collected as a brownish-gray rubbery solid (18.4 mg, 92%).
$^{1}H$ NMR (600 MHz, $CDCl_3$): δ 5.77–5.28 (m, 4H), 4.53–4.27 (br m, 1H), 4.22–3.98 (br m, 1H), 2.53–1.92 (m, 6H).
$^{13}C$ NMR (150 MHz, $CDCl_3$): δ 136.8–123.7, 73.1–70.9, 68.0–66.3, 42.2–39.8, 36.8–34.6, 33.9–31.1, 28.6–26.8.

**Poly(cyclooctane-1,2,3,5,6-pentaol), P2**

In a vial fitted with a magnetic stir bar, dissolve **P1** (95.5 mg, 0.769 mmol, 1.00 equiv) in THF (6.8 mL) and DI water (284 μL). *N*-methylmorpholine *N*-oxide (335 μL, 1.61 mmol, 2.10 equiv, 50 wt% in water) was added to this stirring solution. Osmium(VIII) oxide (235 μL, 38.4 μmol, 0.05 equiv, 4 wt% in water) was added dropwise, and the mixture was stirred open to air for 12 hours at 23 °C. Upon completion, $NaHSO_{3(aq)}$ (5 mL) and $Na_2SO_{3(aq)}$ (5 mL) were added to the stirring reaction mixture. After 10 minutes, the solution was extracted with $CHCl_3$ (10 mL). The remaining aqueous layer was dialyzed (Slide-A-Lyzer™ MINI dialysis device, 3.5K MWCO) with DI water (1000 mL) over 6 hours. The DI water (1000 mL) was then replaced and left to dialyze over a period of 24 hours. The resultant aqueous solution was lyophilized, then further dried in vacuo (~20 mTorr) at 65 °C, yielding a white, fibrous product (121.2 mg, 82.1% yield).
$^{1}H$ NMR (600 MHz, $D_2O$): δ 4.21–3.29 (m, 5H), 2.20–1.36 (m, 6H).
$^{13}C$ NMR (213 MHz, $D_2O$): δ 81.4–68.3, 40.1–35.1, 32.4–29.4.

**General procedure for ROMP of M1 using chain transfer agent (CTA)**

The same procedure was followed for the synthesis of **P1**, but *cis*-4-octene or *cis*-butene diacetate were employed as the CTA (15.6 or 49.3 equiv) and added to the solution of **M1** (30.0 mg, 0.242 mmol, 4000 equiv) in THF prior to **G3** (44.0 μg, 0.0604 μmol, 1.00 equiv) addition.

**Supplementary Figures**

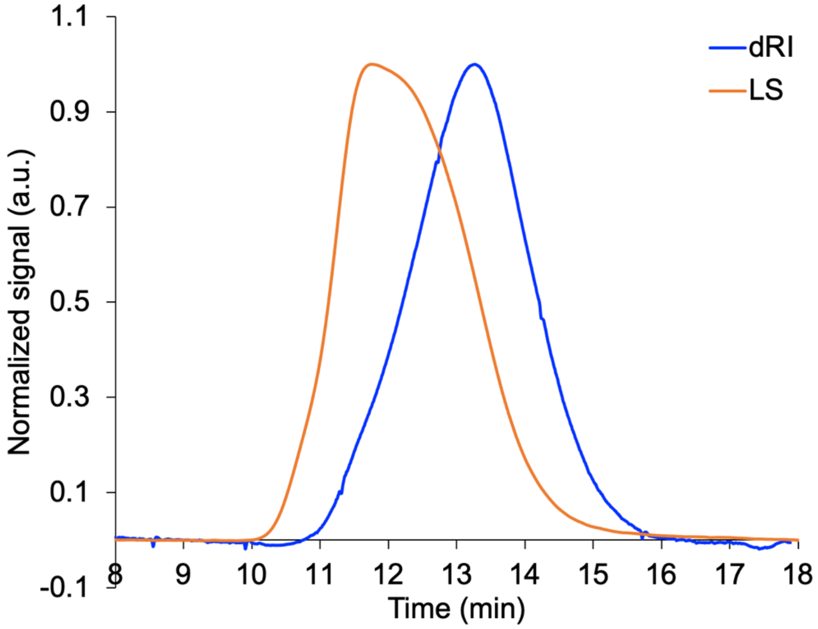


**Figure S1.** GPC traces of differential refractive index (dRI) and light scattering (LS) of **P1**, $M_n$ = 86.7 kg/mol, *Đ* = 2.37.

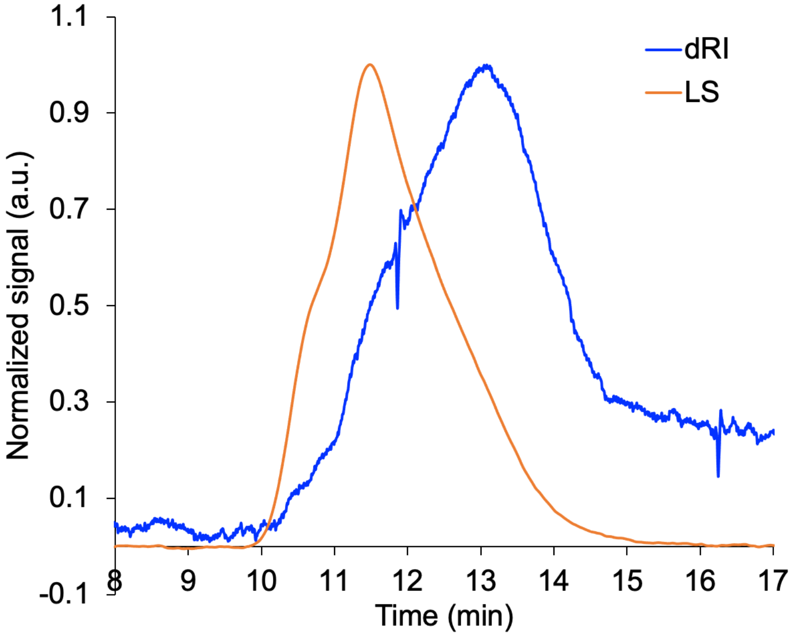


**Figure S2.** GPC traces of differential refractive index trace (dRI) and light scattering (LS) of **P1**, $M_n$ = 163 kg/mol, $Đ$ = 2.41.

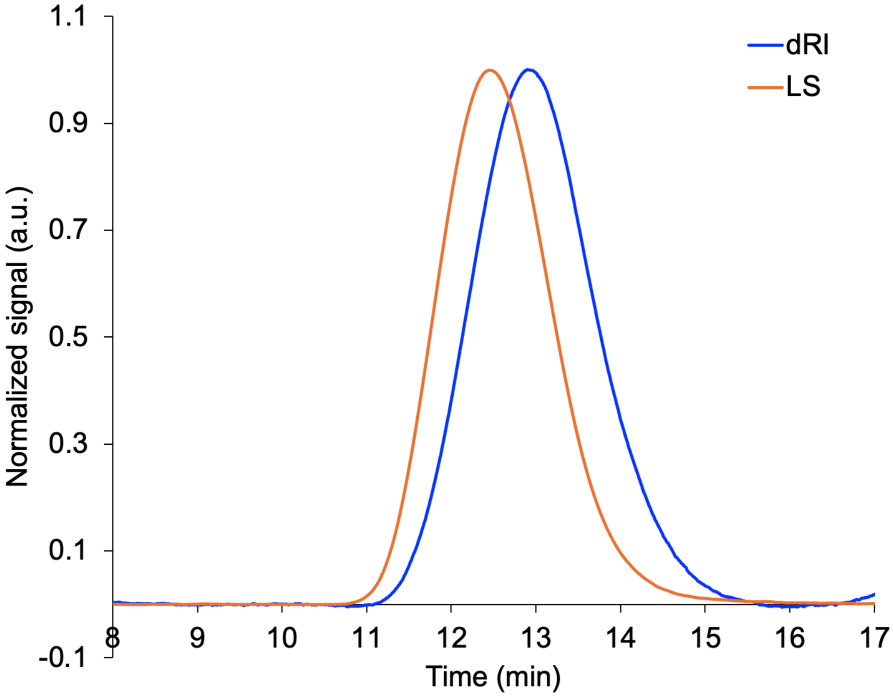


**Figure S3.** GPC traces of differential refractive index (dRI) and light scattering (LS) of **P1**, $M_n$ = 92.2 kg/mol, $Đ$ = 1.57.

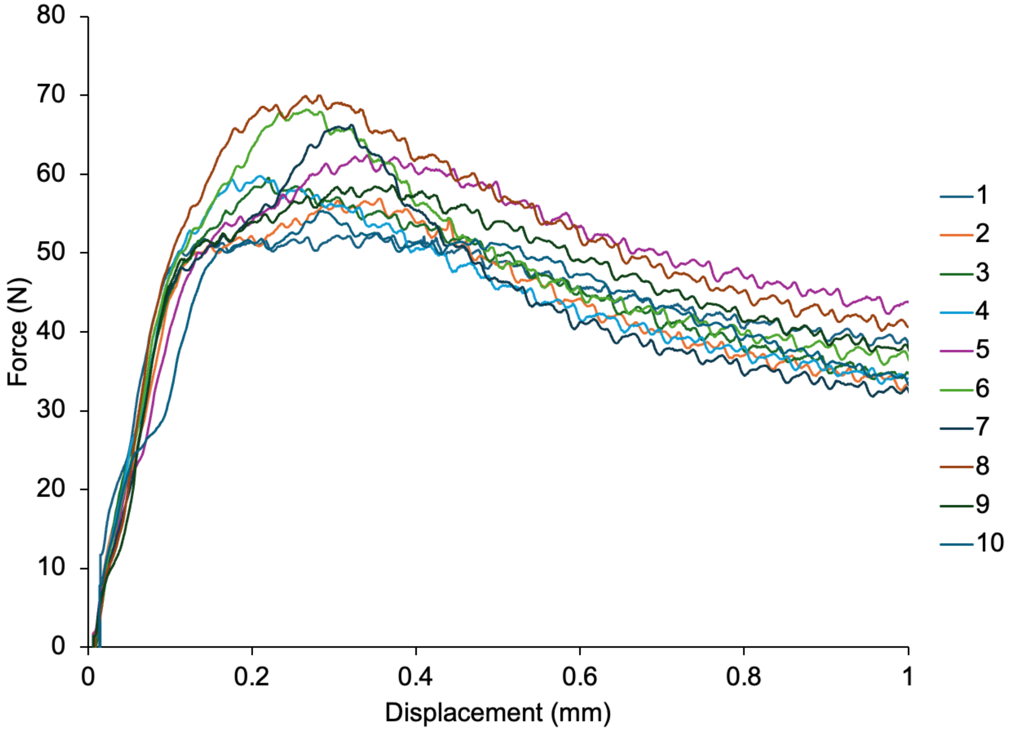

**Figure S4.** Force (N) vs. displacement (mm) data for 10 replicates of **P1** as an adhesive on glass.

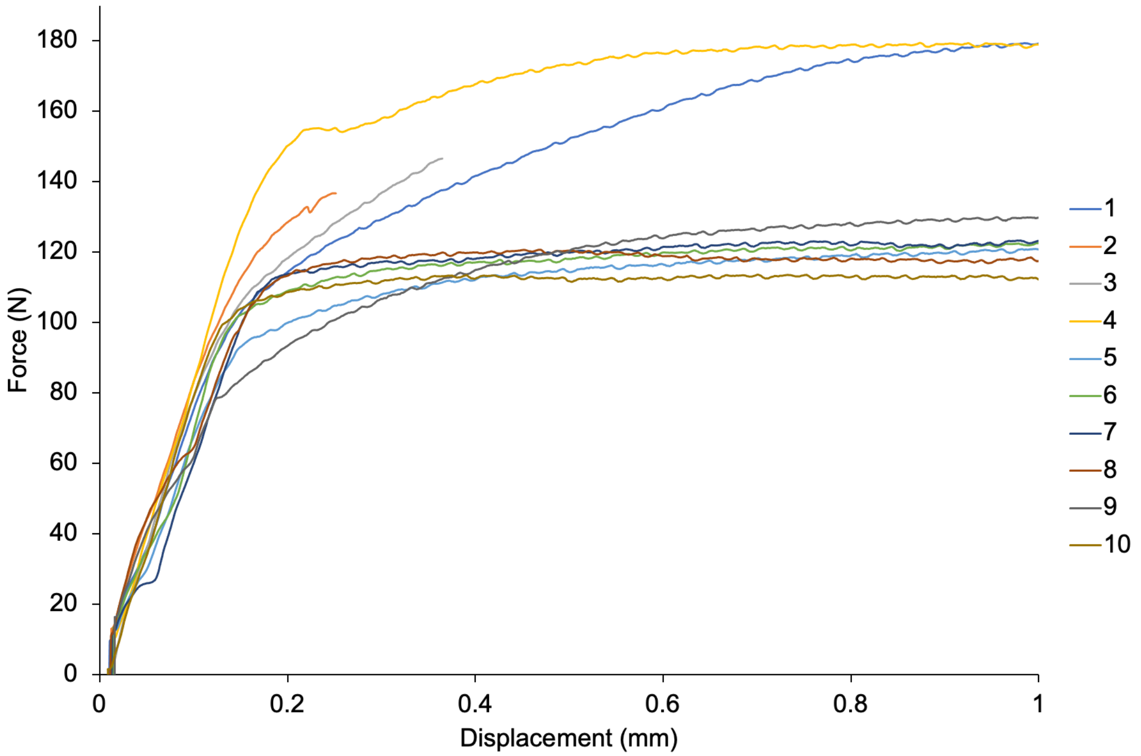


**Figure S5.** Force (N) vs. displacement (mm) data for 10 replicates of **P2** as an adhesive on glass.

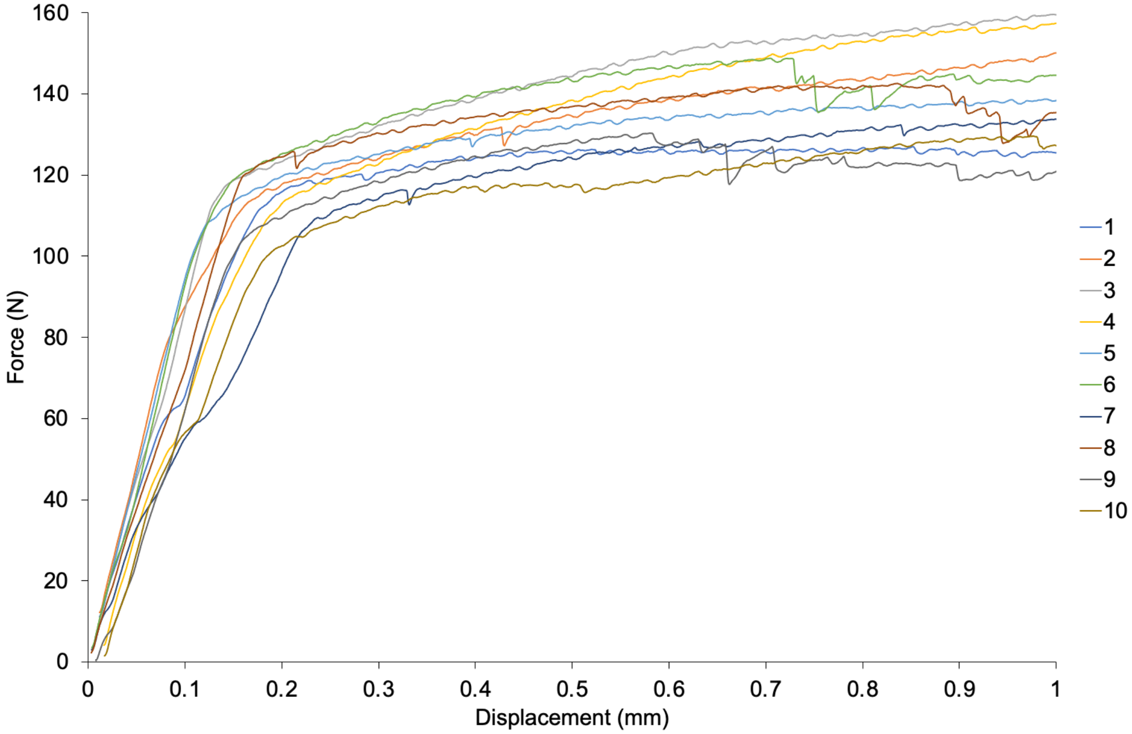


**Figure S6.** Force (N) vs. displacement (mm) data for 10 replicates of Elmer's® clear glue as an adhesive on glass.

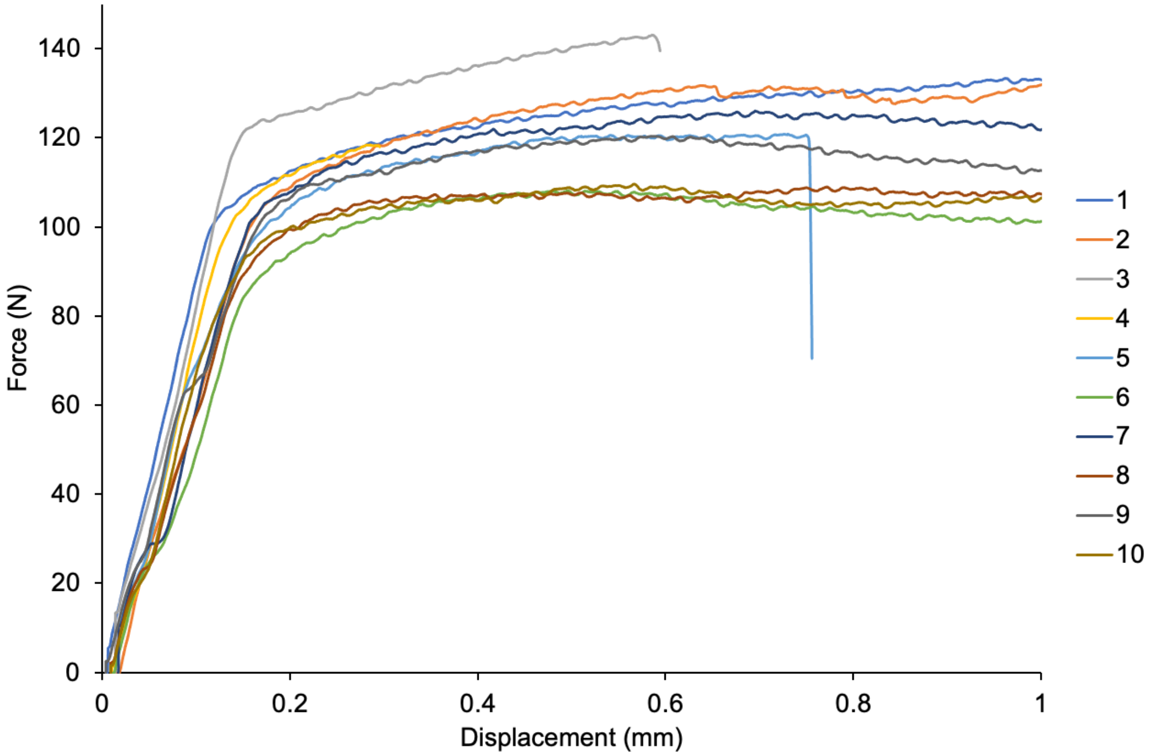


**Figure S7.** Force (N) vs. displacement (mm) data for 10 replicates of PVA as an adhesive on glass.

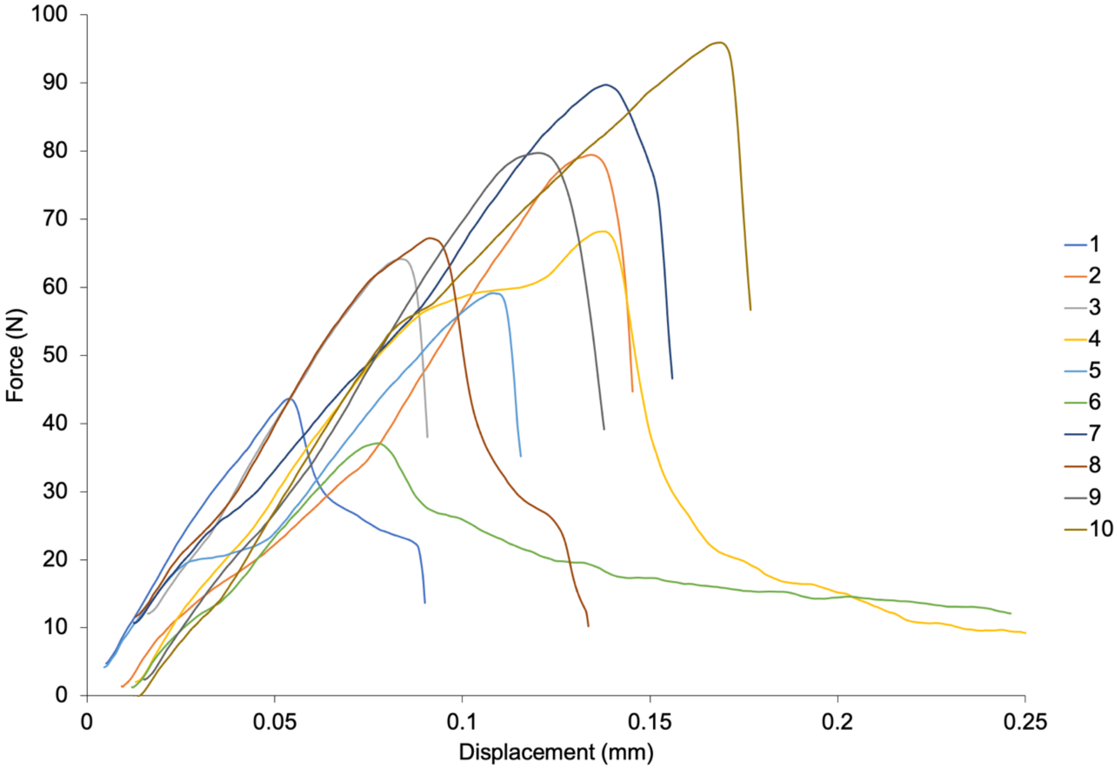


**Figure S8.** Force (N) vs. displacement (mm) data for 10 replicates of PEG as an adhesive on glass.

## Supplementary Tables

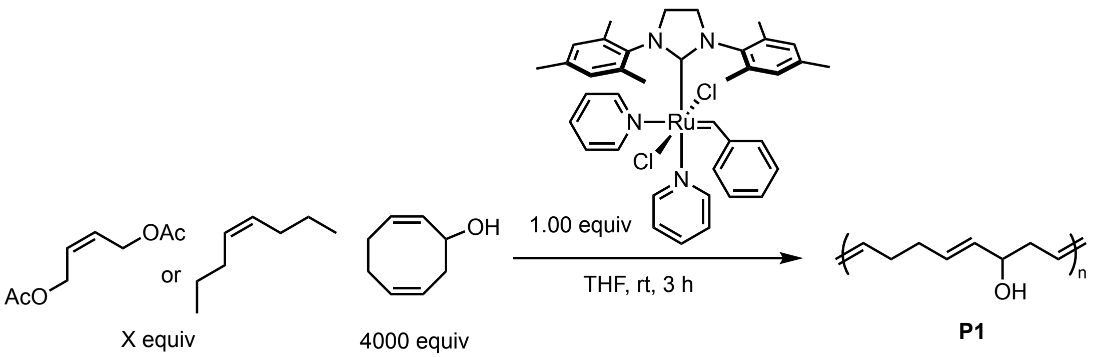


| CTA | X equiv CTA | Time | target $M_n$ (g/mol) | actual $M_n$ (g/mol) | $Đ$ |
|---|---|---|---|---|---|
| *cis*-butene diacetate | 49.3 | 10 min | 10,000 | 26,600 | 1.80 |
| *cis*-butene diacetate | 49.3 | 30 min | 10,000 | 25,400 | 1.52 |
| *cis*-butene diacetate | 49.3 | 1.5 h | 10,000 | 22,900 | 1.58 |
| *cis*-butene diacetate | 49.3 | 3 h | 10,000 | 22,500 | 1.60 |
| *cis*-butene diacetate | 15.6 | 10 min | 30,000 | 69,700 | 1.56 |
| *cis*-butene diacetate | 15.6 | 30 min | 30,000 | 67,700 | 1.46 |
| *cis*-butene diacetate | 15.6 | 1.5 h | 30,000 | 78,100 | 1.35 |
| *cis*-butene diacetate | 15.6 | 3 h | 30,000 | 59,700 | 1.43 |
| *cis*-4-octene | 49.3 | 10 min | 10,000 | 32,800 | 1.38 |
| *cis*-4-octene | 49.3 | 30 min | 10,000 | 31,300 | 1.31 |
| *cis*-4-octene | 49.3 | 1.5 h | 10,000 | 24,800 | 1.38 |
| *cis*-4-octene | 49.3 | 3 h | 10,000 | 24,800 | 1.38 |
| *cis*-4-octene | 15.6 | 10 min | 30,000 | 72,400 | 1.51 |
| *cis*-4-octene | 15.6 | 30 min | 30,000 | 65,300 | 1.40 |
| *cis*-4-octene | 15.6 | 1.5 h | 30,000 | 58,300 | 1.45 |
| *cis*-4-octene | 15.6 | 3 h | 30,000 | 60,400 | 1.41 |

**Table S1.** Molecular weight and dispersity data from various timepoints for ROMP experiments with chain transfer agents.

| **P2** sample number | adhesive weight (mg) | yield strength (N) | yield strength (MPa) |
|---|---|---|---|
| 1 | 2.8 | 104.4 | 0.164 |
| 2 | 1.6 | 112.4 | 0.177 |
| 3 | 1.3 | 96.7 | 0.152 |
| 4 | 1.1 | 141.2 | 0.222 |
| 5 | 1.8 | 90.8 | 0.143 |
| 6 | 1.5 | 101.5 | 0.160 |
| 7 | 1.9 | 110.6 | 0.174 |
| 8 | 1.2 | 110.8 | 0.175 |
| 9 | 1.9 | 93.2 | 0.147 |
| 10 | 2.4 | 101.7 | 0.160 |
| **average** | **1.7** | **106.3** | **0.167** |

**Table S2.** Yield strength and weight data for **P2** replicates.

| Elmer’s® sample number | adhesive weight (mg) | yield strength (N) | yield strength (MPa) |
|---|---|---|---|
| 1 | 6.6 | 111.7 | 0.176 |
| 2 | 8.0 | 114.6 | 0.180 |
| 3 | 5.5 | 118.0 | 0.186 |
| 4 | 9.6 | 110.2 | 0.174 |
| 5 | 6.7 | 119.3 | 0.188 |
| 6 | 7.2 | 115.9 | 0.183 |
| 7 | 4.6 | 109.3 | 0.172 |
| 8 | 7.0 | 120.7 | 0.190 |
| 9 | 7.8 | 103.8 | 0.163 |
| 10 | 8.2 | 100.9 | 0.159 |
| **average** | **7.1** | **112.4** | **0.177** |

**Table S3.** Yield strength and weight data for Elmer’s® clear glue replicates.

| PEG sample number | adhesive weight (mg) | yield strength (N) | yield strength (MPa) |
|---|---|---|---|
| 1 | 1.7 | 43.6 | 0.069 |
| 2 | 2.6 | 79.3 | 0.125 |
| 3 | 2.1 | 64.1 | 0.101 |
| 4 | 2.2 | 56.2 | 0.089 |
| 5 | 2.0 | 19.7 | 0.031 |
| 6 | 0.7 | 37.1 | 0.058 |
| 7 | 2.1 | 89.7 | 0.141 |
| 8 | 1.8 | 67.2 | 0.106 |
| 9 | 1.4 | 79.8 | 0.126 |
| 10 | 1.5 | 95.9 | 0.151 |
| **average** | **1.8** | **63.3** | **0.100** |

**Table S4.** Yield strength and weight data for PEG replicates.

| PVA sample number | adhesive weight (mg) | yield strength (N) | yield strength (MPa) |
|---|---|---|---|
| 1 | 2.5 | 104.8 | 0.165 |
| 2 | 2.4 | 103.1 | 0.162 |
| 3 | 5.6 | 120.3 | 0.189 |
| 4 | 1.3 | 97.8 | 0.154 |
| 5 | 8.2 | 99.6 | 0.157 |
| 6 | 6.6 | 90.1 | 0.142 |
| 7 | 7.9 | 109.2 | 0.172 |
| 8 | 4.5 | 95.7 | 0.151 |
| 9 | 6.0 | 104.0 | 0.164 |
| 10 | 6.9 | 96.8 | 0.152 |
| **average** | **5.2** | **102.1** | **0.161** |

**Table S5.** Yield strength and weight data for PVA replicates.

| **P1** sample number | adhesive weight (mg) | yield strength (N) | yield strength (MPa) |
|---|---|---|---|
| 1 | 8.3 | 48.4 | 0.076 |
| 2 | 7.3 | 48.5 | 0.076 |
| 3 | 7.7 | 47.4 | 0.075 |
| 4 | 7.5 | 48.5 | 0.076 |
| 5 | 7.0 | 49.9 | 0.079 |
| 6 | 6.8 | 46.8 | 0.074 |
| 7 | 7.3 | 45.2 | 0.071 |
| 8 | 7.2 | 46.9 | 0.074 |
| 9 | 7.4 | 47.8 | 0.075 |
| 10 | 3.1 | 48.7 | 0.077 |
| **average** | **7.0** | **47.8** | **0.075** |

**Table S6.** Yield strength and weight data for **P1** replicates.

## Spectral Data

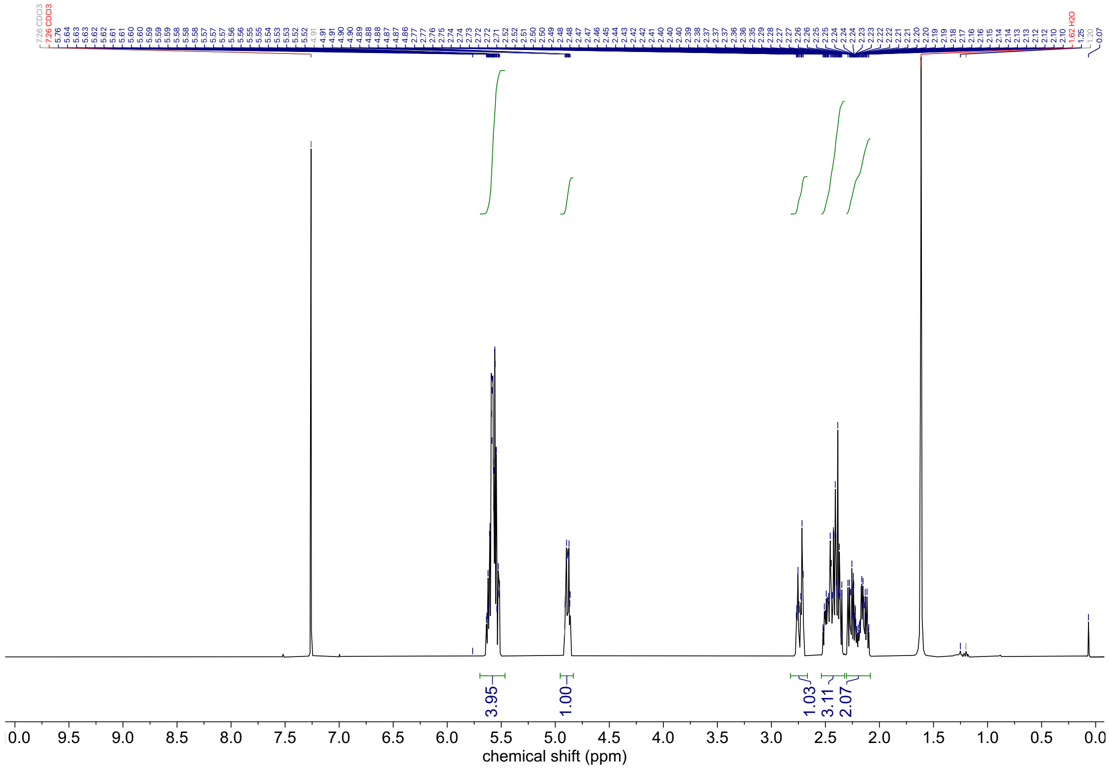

**Figure S9.** $^1$H NMR (400 MHz, $CDCl_3$, 23 °C) of (2*Z*,6*Z*)-cycloocta-2,6-dien-1-ol.

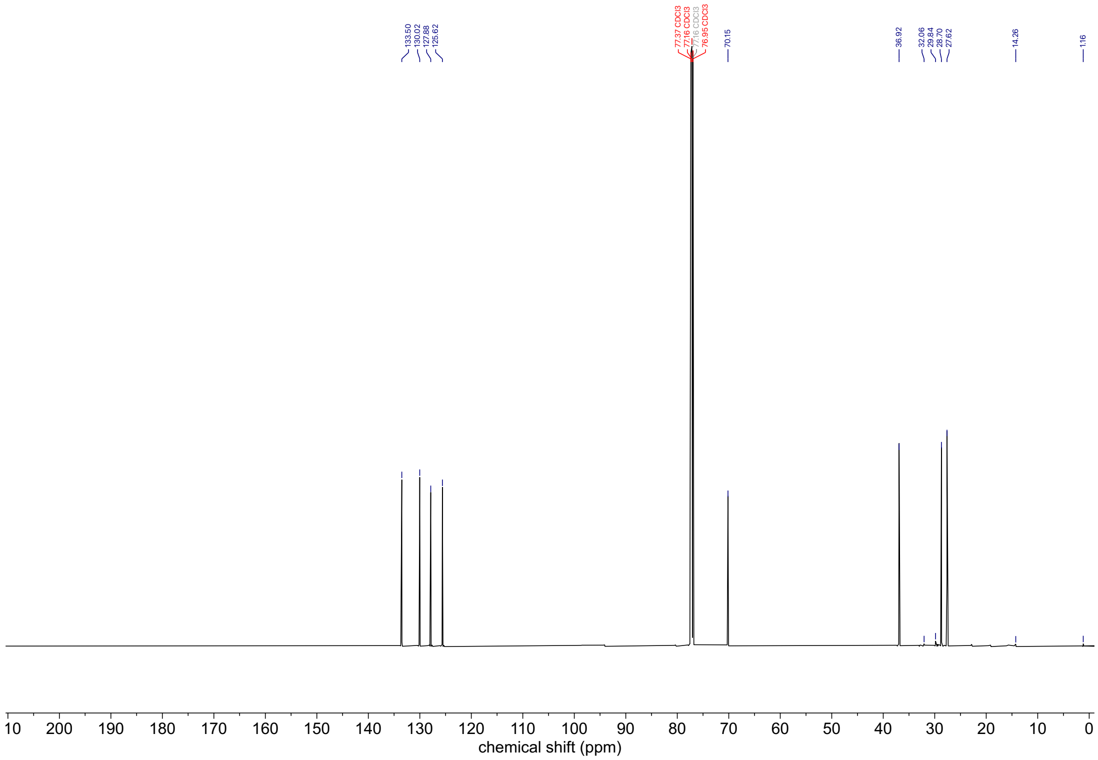


**Figure S10.** $^{13}$C NMR (150 MHz, $CDCl_3$, 23 °C) of (2*Z*,6*Z*)-cycloocta-2,6-dien-1-ol.

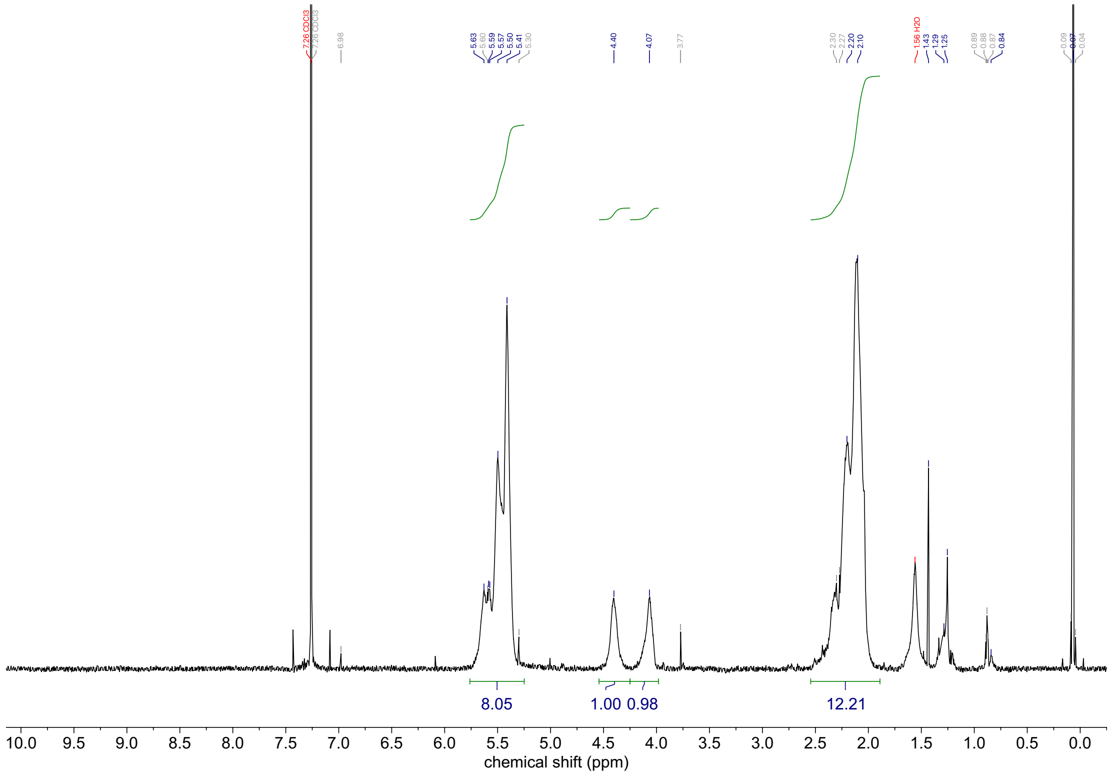


**Figure S11.** $^1$H NMR (600 MHz, $CDCl_3$, 23 °C) of **P1**.

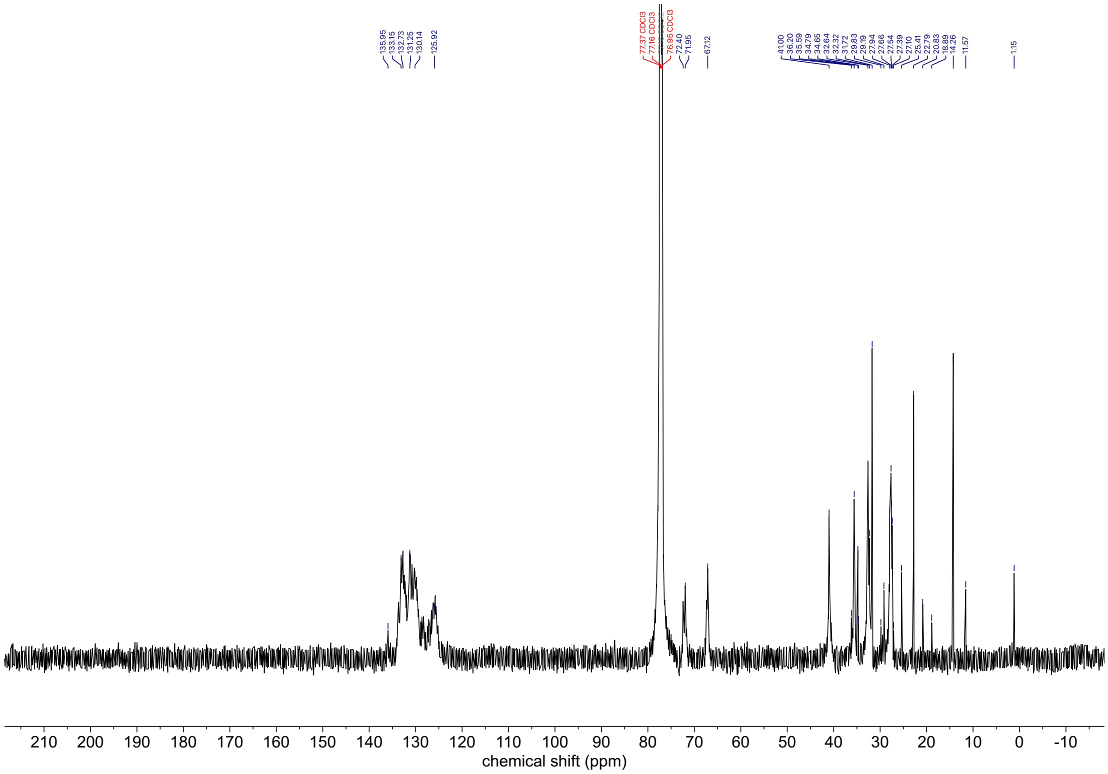

**Figure S12.** $^{13}$C NMR (150 MHz, $CDCl_3$, 23 °C) of **P1**.

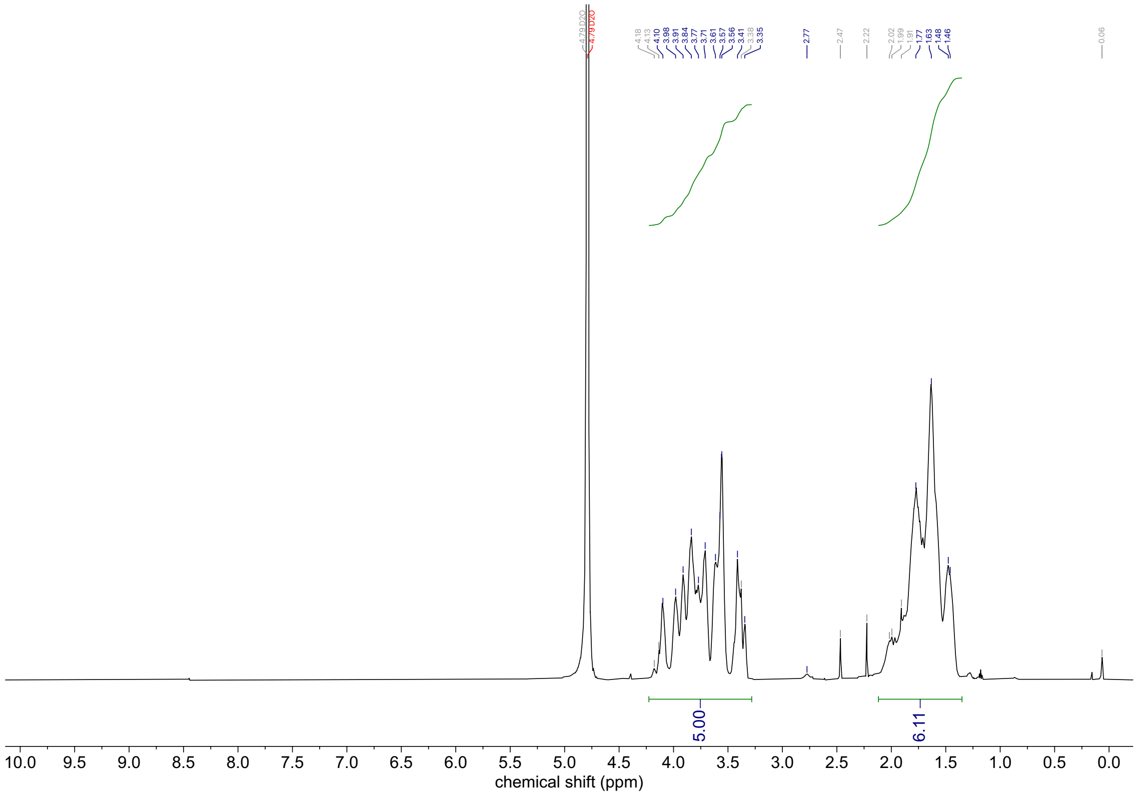


**Figure S13.** $^{1}H$ NMR (600 MHz, $D_2O$, 23 °C) of **P2**.

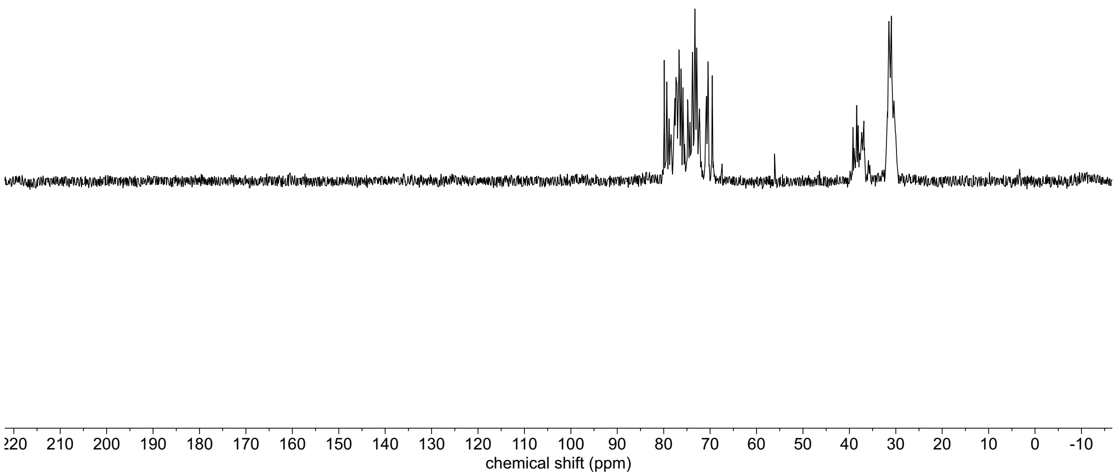


**Figure S14.** $^{13}$C NMR (213 MHz, $D_2O$, 23 °C) of **P2**.

## References


(1) Pangborn, A.B.; Giardello, M.A.; Grubbs, R.H.; Rosen, R. K.; Timmers, F.J. Safe and Convenient Procedure for Solvent Purification. *Organometallics* **1996**, *15* (5), 1518–1520. https://doi.org/10.1021/om9503712.
(2) Fulmer, G.R.; Miller, A.J. M.; Sherden, N.H.; Gottlieb, H.E.; Nudelman, A.; Stoltz, B.M.; Bercaw, J.E.; Goldberg, K.I. NMR Chemical Shifts of Trace Impurities: Common Laboratory Solvents, Organics, and Gases in Deuterated Solvents Relevant to the Organometallic Chemist. *Organometallics* **2010**, *29* (9), 2176–2179. https://doi.org/10.1021/om100106e.
(3) Sanford, M.S.; Love, J.A.; Grubbs, R.H. Mechanism and Activity of Ruthenium Olefin Metathesis Catalysts. *J. Am. Chem. Soc.* **2001**, *123* (27), 6543–6554. https://doi.org/10.1021/ja010624k.
